# R-100 improves pulmonary function and systemic fluid balance in sheep with combined smoke-inhalation injury and *Pseudomonas aeruginosa* sepsis

**DOI:** 10.1186/s12967-017-1366-6

**Published:** 2017-12-28

**Authors:** Hiroshi Ito, Erik Malgerud, Sven Asmussen, Ernesto Lopez, Andrew L. Salzman, Perenlei Enkhbaatar

**Affiliations:** 1 Department of Anesthesiology, The University of Texas Medical Branch, Galveston, TX USA; 2 Radikal Therapeutics, Inc., West Tisbury, MA USA

**Keywords:** Sepsis, Redox, R-100, Smoke

## Abstract

**Background:**

Septic shock is a major cause of death in intensive care units around the world . The aim of the study was to investigate whether the novel drug R-100 (a superoxide degradation catalyst and nitric oxide donor) improves pulmonary function in a sheep model of septic shock caused by *Pseudomonas aeruginosa* and smoke inhalation.

**Methods:**

Eleven female sheep were prepared surgically and randomly assigned to a treatment group (n = 5) or a control group (n = 6) after inhalation of cooled cotton smoke and airway instillation of live *P. aeruginosa* (2.5 × 10^11^ CFU) by bronchoscope under deep anesthesia and analgesia. The treatment group received an intravenous infusion of a total of 80 mg/kg of R-100 diluted in 500 mL of 5% dextrose. The control group was given 500 mL of 5% dextrose. All animals received intravenous lactated Ringer’s solution to maintain a hematocrit level at baseline ± 3%. Blood gas and hemodynamics were measured at baseline and then analyzed every 3 h during the 24-h study period. Results are expressed as mean ± SEM.

**Results:**

The treated animals showed significant improvement in their pulmonary gas exchange (PaO_2_/FiO_2_ ratio at 24 h: 246 ± 29 vs. 90 ± 40 mmHg control, P < 0.05). Pulmonary arterial pressures were reduced in the treated group (24 h: 26 ± 1 vs. 30 ± 2 cm mmHg control, P < 0.05). The treated animals also had an improved total fluid balance after 24 h (190 ± 45/24 h mL vs. 595 ± 234/24 h mL control, P < 0.05).

**Conclusions:**

Treatment with R-100 improves pulmonary gas exchange and blood oxygenation, and prevents a fluid imbalance in sheep subjected to smoke inhalation and *P. aeruginosa*.

## Background

It is well known that patients with acute lung injury (ALI), who develop sepsis have a poor prognosis [1, 2]. In ALI-mediated sepsis, reactive oxygen species, such as superoxide are released as an effect of a massive inflammatory response [3, 4].

Oxidative stress is often implicated in the development of vascular dysfunction [5]. Under normal circumstances, superoxide is degraded by superoxide dismutase (SOD). However, during a forceful activation of neutrophils, as seen in sepsis, SOD becomes saturated and the level of superoxide becomes elevated [6–8]. An excess superoxide production can, aside from damaging endothelial cells, react nonenzymatically with nitric oxide (NO) to yield the more toxic product peroxynitrite (ONOO^−^), which causes vascular hyper-permeability and tissue damage [9–11].

Although excessive NO, especially iNOS-derived, is generally regarded as detrimental in sepsis pathophysiology, studies have instead shown that non-selective inhibition of NO production may cause pulmonary hypertension [12]. It is worth noting that increased pressure in pulmonary circulation during sepsis is associated with a reduced patient survival [13]. Numerous studies besides our own have shown that administration of inhaled NO may normalize increased pulmonary arterial pressure [14–16].

The aim of this study was to investigate the effects of simultaneous delivery of NO while limiting peroxynitrite formation by catalytically degrading superoxide. Promoting degradation of accumulated superoxide decreases vascular injury in sepsis by inhibiting production of peroxynitrite and improves pulmonary function by increasing the availability of NO in the pulmonary circulation. This pathway yields a potential therapeutic target in ALI and sepsis.

Currently, there are no studies investigating the combined effects of superoxide catalytic degradation and simultaneous augmentation of nitric oxide availability. R-100 (Radical Therapeutics, Inc., West Tisbury, MA) is a multifunctional redox active small molecule, comprising two distinct moieties: (1) an organic nitrate domain that donates nitric oxide, and (2) a nitroxide domain that serves as a redox degradation catalyst and removes superoxide anions and hydrogen peroxide. The fusion of both moieties into a single molecular entity allows for the donation of nitric oxide without concomitant generation of peroxynitrite via the diffusion-limited reaction of the donated NO with ambient superoxide anion. The administration of R-100 in septic sheep would hypothetically remove superoxide from the circulating blood, thus indirectly inhibiting production of peroxynitrite leading to an attenuation of vascular injury. Secondly, NO supplementation would be expected to decrease pulmonary circulatory pressures, decrease pulmonary shunt fraction and improve blood oxygenation. In the current study, the objective was to test these hypotheses using R-100 in a well-characterized ovine model of sepsis/septic shock.

## Methods

Eleven adult female Merino sheep were surgically prepared and subjected to smoke inhalation injury followed by installation of *Pseudomonas aeruginosa* into the lungs. This study was approved by the Institutional Animal Care and Use Committee of the University of Texas Medical Branch and conducted in compliance with the guidelines of the National Institutes of Health and the American Physiology Society for the care, handling, and use of laboratory animals. The studies were completed at the institution’s Translational Intensive Care Unit, which is a facility accredited by the Association for the Assessment and Accreditation of Laboratory Animal Care.

### Surgical preparation

The eleven sheep, weighing 35 ± 0.5 kg were surgically prepared for the experimental procedure. After induction of anesthesia with ketamine (Bioiche Pharma, Lake Forest, IL, 15–20 mg/kg) and under isoflurane anesthesia, a 16 G, 24 inch silastic catheter was inserted into the right femoral artery (Intracath, Becton–Dickinson, Sandy, UT, USA). A pulmonary arterial thermodilution catheter, model 131F7 was also positioned in the pulmonary artery through the right common jugular vein (Edwards Lifesciences LLC, Irvine, CA, USA). A silastic (0.062 inch ID) catheter was also inserted into the left atrium through a left thoracotomy in the fifth intercostal space (Dow Corning, Midland, MI, USA). Pre-emptive analgesia was provided with buprenorphine. Postoperative analgesia was maintained with continuous infusion of buprenorphine over 48 h and thereafter as needed.

### Experimental procedure

After 5 days of recovery with free access to food and water, tracheostomy (Shiley 10 SCT, Tyco Healthcare, Plesanton, CA) was performed under ketamine/isoflurane anesthesia and analgesia. Pneumonia and sepsis were induced by smoke inhalation and instillation of live *P. aeruginosa* into the lungs. Briefly, a modified bee smoker was filled with 40 g of smoke from cotton toweling, and attached to the tracheostomy tube via a modified endotracheal tube containing an indwelling thermistor from a pulmonary arterial catheter to monitor the temperature of the smoke. Four sets of 12 breaths of cotton smoke (< 40 °C) were manually delivered. After each set, the arterial carboxyhemoglobin (COHb) level was measured with a 626 CO-Oximeter (Instrumentation Laboratory, Bedford, MA, USA) to ensure that all sheep received a similar level of injury. Immediately after the smoke injury, an amount of 2.5 × 10^11^ Colony Forming Units (CFU) of live *P. aeruginosa* (PD-05144, 27317, ATCC) suspended in 30 mL of saline was instilled into the lungs through a bronchoscope. 20 mL were placed into the right lung (10 mL in the main bronchus of the middle lobe, and 10 mL in the main bronchus of the lower lobe) and 10 mL were instilled into the main bronchus of the left lower lobe, thus compensating for the extra lobe of the right lung. A urinary retention catheter (Foley 14 C.R., Bard Inc., Covington, GA) was placed into the urinary bladder via the urethra as well.

After this, a randomization occurred to either treatment with R-100 (n = 5) or placebo (n = 6). The experiment was conducted with two sheep being studied simultaneously side-by-side. Treatment was started 1 h post-injury. The treatment group received R-100 at a dose of 80 mg/kg in 500 mL of 5% dextrose. A bolus (30 min) of 10 mg/kg was followed by the remaining 70 mg/kg as a continuous intravenous infusion over 24 h. The control group was treated with an equivalent amount of 5% dextrose. R-100 was kindly provided by Radikal Therapeutics, Inc. Dosage was based on previous mice studies results (Unpublished data). Mice were given 20, 40 or 80 mg/kg/day of intraperitoneal injection of R-100, which was started 1 h after LPS challenge. Although the severity of injury was attenuated with 20, 80 mg/kg had the most survival benefit (100% 7-day survival, while none of mice survived in non-treated control group). None of the animal groups received antibiotics before (at least 24 h) or during the experiment.

After the injury, the animals were awakened and maintained on mechanical ventilation (Servo Ventilator 300C, Siemens-Elema, Sweden) with a positive end-expiratory pressure (PEEP) of 5 cmH_2_O and a tidal volume of 12 mL/kg [17] during the 24 h experimental period.

FiO_2_ was adjusted after 1 h and then every 3 h according to arterial blood gas analysis results to, as far as possible, maintain the arterial SaO_2_ > 90% and PaO_2_ at baseline (~ 100 mmHg). The respiratory rate was also adjusted to keep PaCO_2_ between 30 and 40 mmHg. Peak and plateau airway pressures were monitored throughout the experimental period. The animals received fluid resuscitation (Ringer’s lactate, containing sodium 130 mmol/L, chloride 109 mmol/L, lactate 28 mmol/L, potassium 4 mmol/L, calcium 1.5 mmol/L), initially at a rate of 2 mL/kg/h, which was then adjusted to maintain hematocrit at baseline ± 3%. The maximum fluid infusion rate allowed was 15 mL/kg/h.

Plasma and urine samples were taken every 3 h during the 24-h experimental period. All sheep were euthanized at 24 h after the injury.

### Measurements

Mean arterial pressure (MAP), left atrial pressure (LAP), central venous pressure (CVP), pulmonary artery pressure (PAP), pulmonary capillary wedge pressure (PCWP) and heart rate (HR) were measured using pressure transducers (model P × 3 × 3; Baxter Edwards Critical Care Division, Irvine, CA) connected to a hemodynamic monitor (IntelliVue MP50; Philips Medizin Systeme Boeblingen, Boeblingen, Germany). Cardiac output (CO) was determined by standard thermodilution method. Stroke volume index (SVI), systemic vascular resistance index (SVRI), pulmonary vascular resistance index (PVRI), left ventricle stroke work index (LVSWI), and right ventricle stroke volume index (RVSWI) were calculated by standard formula.

Arterial and mixed venous blood was drawn at baseline and then every 3 h for blood gas measurements by an analyzer (Premier 300, Instrumental Laboratory, Lexington, MA) and the results were corrected for body temperature.

Urine output and fluid administration were measured at regular time intervals for calculation of fluid balance. Plasma protein concentration was measured by a refractometer.

The airway peak and plateau pressures were recorded from ventilator readouts. The bloodless lung wet-to-dry weight ratio was calculated according to the equation described by Pearce et al. [18].

Pulmonary shunt fraction (Qs/Qt), PaO_2_/FiO_2_ ratio, and systemic and pulmonary vascular resistance indexes were calculated according to standard formula.

Histological analysis was performed on tissue samples taken from the trachea, bronchioles, and alveoli. Scoring was performed for airway congestion, septal edema, alveolar edema, and alveolar polymorphonuclear neutrophils (PMNs) according to a previously established scoring system [19].

### Statistical analysis

The data were compared using analysis of variance (ANOVA) for repeated measures. Bonferroni post hoc tests were performed for comparisons between groups at each time point, and student’s t-tests were performed when appropriate. All values are expressed as mean ± standard error of the mean (SEM). The statistical analysis was performed using GraphPad Prism Software (GraphPad Software, La Jolla, CA, USA). P-values < 0.05 were considered significant.

## Results

### Baseline variables

There were no differences between the two study groups at baseline within the measured variables. Body weight was similar in both groups (35.4 ± 0.8 kg in the treatment group vs. 35.2 ± 0.9 kg in the control), and there were no differences in body surface area between the groups (0.91 ± 0.01 m^2^ in the treatment group vs. 0.91 ± 0.01 m^2^ in the control). The arterial COHb determined immediately after the smoke-inhalation injury averaged 69.1 ± 2.0% in the control group and 64.4 ± 2.5% in the R-100 group; these levels were not significantly different from each other indicating a similar degree of injury. The core body temperatures similarly increased in both groups and remained comparable. Lactate levels after 3 h were 3.4 ± 1.1 mg/dL in the R-100 group and 2.9 ± 1.0 mg/dL in the control group, indicating tissue hypoxia in both groups. These values rose throughout the experiment and there were no differences between the groups.

### Pulmonary gas exchange and global oxygen transport

**Fig. 1:**
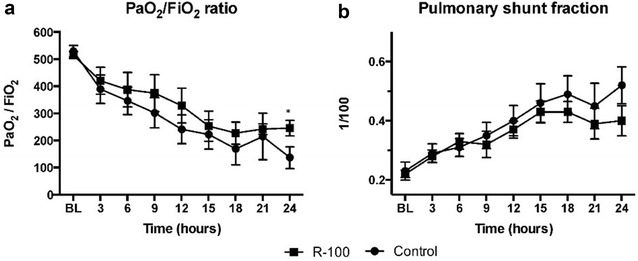
Effects of R-100 on pulmonary gas exchange evaluated by PaO_2_/FiO_2_ ratio (**a**) and pulmonary shunt fraction (**b**). PaO_2_ is a oxygen partial pressure (mm Hg) in arterial blood. FiO_2_ represents oxygen partial pressure (mm Hg) in inspired air. Data are expressed as mean ± SEM. *P < 0.05 vs. control

The PaO_2_/FiO_2_ (P/F) ratio declined in both animal groups, however at 24 h the R-100 group had a significantly higher P/F ratio than the control group (P < 0.05) (Fig. 1a). The pulmonary shunt fraction (Qs/Qt) increased in both groups implying that the fraction of the blood that was shunted past the lungs was higher as the experiment proceeded. The groups did not differ from each other with respect to mean Qs/Qt means (Fig. 1b).

Comparisons between the groups with respect to PaCO_2_, SvO_2_, PvO_2_, and PvCO_2_ did not show any significant differences. As mentioned, the FiO_2_ was adjusted to keep SaO_2_ above 90%. It was maintained above the targeted value throughout the study period; however, the SaO_2_ in control sheep remained below 90% even with maximum increased FiO_2_ (100%). At 24 h, SaO_2_ was significantly higher in the R-100 sheep compared to control (Table 1).

**Table 1:** Hemodynamic data

	BL	3 h	6 h	9 h	12 h	15 h	18 h	21 h	24 h
CO									
Control	5.37 ± 0.4	5.87 ± 0.59	5.76 ± 0.39	6.16 ± 0.44	5.93 ± 0.52	5.82 ± 0.47	6.19 ± 0.93	7.38 ± 0.65	7.53 ± 1.52
R-100	5.58 ± 0.24	6.07 ± 0.26	6.16 ± 0.36	6.63 ± 0.41	6.99 ± 0.27	6.93 ± 0.26	6.81 ± 0.39	6.62 ± 0.41	6.63 ± 0.32
MAP									
Control	99 ± 1.8	106 ± 4.7	95 ± 6.5	88 ± 7.6	78 ± 9.6	74 ± 10.0	72 ± 10.7	81 ± 8.6	83 ± 7.9
R-100	95 ± 3.2	99 ± 6.7	103 ± 9.5	98 ± 9.0	84 ± 9.5	76 ± 11.4	73 ± 11.9	85 ± 10.4	84 ± 6.9
HR									
Control	85 ± 2	97 ± 6	114 ± 6	116 ± 7	112 ± 8	115 ± 7	116 ± 8	123 ± 8	122 ± 13
R-100	74 ± 3	109 ± 8	136 ± 11	136 ± 10	141 ± 9	134 ± 8	113 ± 8	116 ± 7	114 ± 7
PAP									
Control	19.5 ± 0.8	23.3 ± 0.4	23.7 ± 1.1	25.3 ± 0.5	26.0 ± 1.1	26.2 ± 1.4	27.8 ± 1.4	29.6 ± 0.7	30.2 ± 1.9*
R-100	19.0 ± 0.5	19.8 ± 1.1	21.0 ± 1.4	22.4 ± 0.9	24.6 ± 0.7	24.0 ± 1.1	24.6 ± 0.7	25.4 ± 0.8	25.6 ± 1.0
PCWP									
Control	10.2 ± 0.7	14.3 ± 0.9	14.3 ± 1.0	15.8 ± .7	16.5 ± 0.6	16.2 ± 0.8	16.5 ± 0.9	17.0 ± 1.4	17.0 ± 2.0
R-100	10.2 ± 0.5	13.2 ± 1.3	13.6 ± 1.4	14.4 ± 0.6	14.2 ± 0.4	13.6 ± 0.4	14.4 ± 0.6	15.0 ± .7	14.8 ± 0.7
CVP									
Control	5.8 ± 0.6	8.2 ± 0.8	9.0 ± 0.9	11.5 ± 1.0	11.3 ± 0.8	11.3 ± 0.5	12.0 ± 1.0	12.2 ± 1.7	11.2 ± 2.3
R-100	5.8 ± 1.0	7.8 ± 1.0	7.8 ± 0.7	9.2 ± 0.5	9.2 ± 1.5	12.0 ± 0.8	13.0 ± 0.6	11.0 ± 0.5	10.8 ± 0.7
LAP									
Control	9.3 ± 0.4	9.8 ± 0.6	10.6 ± 1.0	13.4 ± 0.9	11.6 ± 0.7	12.0 ± 0.6	12.6 ± 1.3	14.8 ± 1.7	12.0 ± 2.9
R-100	8.2 ± 0.4	9.0 ± 1.1	9.2 ± 0.9	11.2 ± 0.5	12.2 ± 0.6	12.4 ± 0.8	13.6 ± 0.7	12.8 ± 0.2	13.0 ± 0.3
SVI									
Control	70.0 ± 6.1	70.5 ± 11.9	57.4 ± 6.2	61.2 ± 8.3	60.2 ± 6.1	57.1 ± 5.5	60.0 ± 9.4	67.0 ± 7.7	69.0 ± 13.3
R-100	84.0 ± 3.3	62.8 ± 5.7	50.5 ± 2.0	54.3 ± 3.5	55.3 ± 3.2	57.9 ± 3.2	67.1 ± 4.4	63.0 ± 1.7	64.5 ± 1.8
SVRI									
Control	1286 ± 93	1259 ± 135	1097 ± 125	922 ± 133	858 ± 166	812 ± 164	776 ± 164	727 ± 150	847 ± 222
R-100	1159 ± 39	1093 ± 56	1134 ± 106	987 ± 124	779 ± 87	683 ± 130	645 ± 119	819 ± 105	798 ± 66
PVRI									
Control	128.0 ± 13.5	117.2 ± 16.5	120.9 ± 17.2	114.5 ± 127	117.7 ± 136	128.8 ± 247	160.9 ± 441	128.0 ± 19	153.0 ± 33
R-100	115.8 ± 12.4	80.5 ± 11.6	87.2 ± 16.3	88.1 ± 6.6	108.2 ± 7.8	109.4 ± 11	110.0 ± 9.0	117.2 ± 6.4	119.1 ± 9.0
LVSWI									
Control	85.0 ± 8.1	90.6 ± 20.2	62.4 ± 8.1	59.4 ± 9.4	51.0 ± 9.3	43.7 ± 7.4	45.3 ± 10.2	57.0 ± 7.5	60.0 ± 10.8
R-100	97.0 ± 6.8	73.9 ± 8.6	61.4 ± 5.6	61.1 ± 5.7	52.7 ± 7.4	48.5 ± 6.8	55.8 ± 13.9	61.2 ± 10.2	60.7 ± 6.4
RVSWI									
Control	12.9 ± 1.1	14.8 ± 2.9	11.3 ± 0.9	11.3 ± 1.2	12.2 ± 1.8	11.7 ± 1.7	12.3 ± 1.5	15.0 ± 1.2	17.0 ± 2.6
R-100	15.0 ± 1.4	10.1 ± 1.0	9.0 ± 0.6	9.7 ± 0.7	11.7 ± 1.5	9.4 ± 1.3	10.8 ± 1.5	12.4 ± 1.0	13.1 ± 1.2
PaCO2									
Control	37.8 ± 0.9	25.6 ± 2.4	27.3 ± 2.4	26.4 ± 2.7	27.8 ± 1.8	26.7 ± 2.7	29.3 ± 2.5	26.6 ± 0.9	36.2 ± 2.6
R-100	36.6 ± 1.2	25.4 ± 1.8	26.6 ± 2.9	27.4 ± 1.0	27.2 ± 1.0	27.0 ± 0.7	25.4 ± 1.6	29.6 ± 1.5	28.4 ± 2.5
SaO2									
Control	91.5 ± 0.3	91.6 ± 2.7	90.2 ± 1.2	90.3 ± 0.6	88.3 ± 1.0	88.4 ± 3.4	87.8 ± 2.6	89.2 ± 2.5	76.2 ± 6.8*
R-100	91.4 ± 0.3	93.8 ± 0.3	90.0 ± 1.1	90.2 ± 0.8	89.6 ± 1.0	88.5 ± 1.3	90.3 ± 1.3	91.7 + 1.4	91.9 ± 0.5
PvO2									
Control	47.3 ± 2.8	47.0 ± 4.2	47.0 ± 4.0	47.8 ± 2.4	46.3 ± 3.5	53.2 ± 3.2	52.3 ± 4.5	54.8 ± 4.8	46.8 ± 6.4
R-100	43.8 ± 2.4	54.4 ± 7.4	51.8 ± 5.3	51.0 ± 1.8	52.4 ± 4.2	52.8 ± 3.9	56.6 ± 2.5	58.0 ± 5.13	60.6 ± 3.5
PvCO2									
Control	44.5 ± 2.3	32.7 ± 2.7	32.5 ± 2.4	30.8 ± 3.3	30.7 ± 1.6	28.7 ± 1.9	32.5 ± 2.4	32.4 ± 1.3	41.2 ± 3.9
R-100	41.4 ± 2.0	33.4 ± 3.3	30.2 ± 1.7	32.6 ± 1.2	31.4 ± 1.3	30.4 ± 1.3	30.6 ± 2.5	35.2 ± 3.3	30.8 ± 2.2
SvO2									
Control	63.6 ± 3.0	65.2 ± 4.6	63.9 ± 3.4	66.6 ± 2.0	63.8 ± 4.0	70.2 ± 4.9	68.9 ± 4.7	69.9 ± 2.7	50.7 ± 8.1
R-100	61.4 ± 1.8	71.3 ± 2.2	67.9 ± 2.4	64.8 ± 3.7	67.0 ± 3.3	68.3 ± 3.2	71.0 ± 4.2	69.4 ± 3.8	72.3 ± 3.5

Data are expressed as mean ± SEM

* P < 0.05 vs. control

**Fig. 2:**
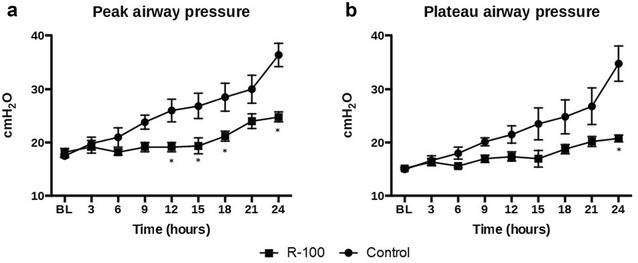
Effects of R-100 on airway peak (**a**) and pause (**b**) pressures. Data are expressed as mean ± SEM. *P < 0.05 vs. control

The peak airway pressure increased in both groups vs. baseline. However, the R-100 group had a significantly lower peak airway pressure than the control group during the last 12 h of the experiment (P < 0.05). The pause airway pressure also increased in both groups, but was lower in the R-100 group (P < 0.05 at 24 h) (Fig. 2).

### Cardiopulmonary hemodynamics

Comparisons between the groups with respect to heart rate, cardiac output, central venous pressure, pulmonary capillary wedge pressure left atrial pressure (increased in both groups vs. baseline) and mean arterial blood pressure and systemic vascular resistance index (decreased in both groups vs. baseline) showed no significant differences.

Pulmonary artery pressure increased throughout the experiment in both groups compared to baseline. However, at 24 h after injury, the R-100 sheep had a significantly lower pulmonary arterial pressure than the control sheep (P < 0.05) (Table 1).

### Fluid balance and plasma protein content

**Fig. 3:**
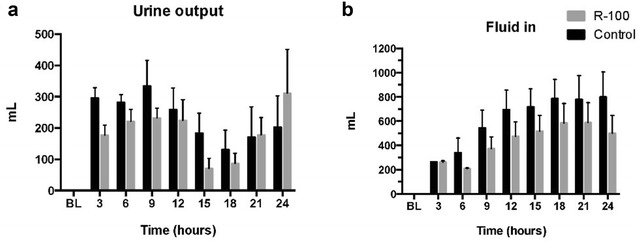
Effects of R-100 on fluid output (urinary output) (**a**) and fluid input (intravenous lactated Ringer’s solution) (**b**). Data are expressed as mean ± SEM

Arterial hemoglobin and hematocrit levels were stable in both groups throughout the study period. Urine output declined in both groups between hours 12 and 18. The R-100 treated-animals appeared to excrete urine at a higher rate than the control group at 24 h, but the difference was not significant (Fig. 3a). The treated group required less fluid to maintain the hematocrit (Fig. 3b).

**Fig. 4:**
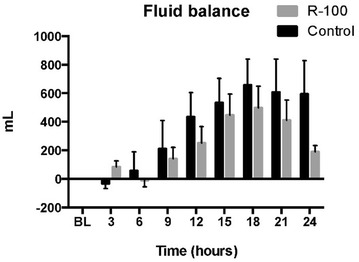
Effects of R-100 on total body fluid accumulation over time. Data are expressed as mean ± SEM. *P < 0.05 vs. control

The body fluid accumulation tended to be higher in the control group throughout the study period. The vehicle-treated control sheep retained 595 ± 234 mL of fluid between 21 and 24 h vs. 190 ± 45 mL in R-100-treated sheep. A significant difference was noted at 24 h between the two groups (Fig. 4).

The post-mortem wet-to-dry weight ratio was slightly higher in the control group. However no significant differences were found between the groups (6.54 ± 0.53 in the control group vs. 6.03 ± 0.70 in the treatment group).

### Histological analysis

Histological analysis did not show any significant differences between the two groups. However, the treatment group tended to having lower scores for all variables, including congestion, septal edema, alveolar edema, and alveolar polymorphonuclear neutrophils (Table 2).

**Table 2:** Histological data

	Congestion	Septal edema	Alveolar edema	Alveolar PMN
R-100	0.70 ± 0.35	0.40 ± 0.23	0.95 ± 0.37	0.35 ± 0.35
Control	1.50 ± 0.6	0.58 ± 0.21	1.92 ± 0.49	1.29 ± 0.43

Data are expressed as mean ± SEM

* P < 0.05 vs. control

## Discussion

The main purpose of this large animal study was to investigate the combined effects of superoxide catalytic degradation and augmentation of nitric oxide by the novel agent R-100 using our well-characterized ovine model of severe sepsis. In the present study the treatment with R-100 limited increases in pulmonary arterial pressure, improved pulmonary gas exchange and reduced airway pressures in septic sheep.

Beneficial effects of the inhibition or reduction of oxidative stress in sepsis has been widely investigated [19–21]. Endothelial tissue injury caused by these reactive oxygen species is classically associated with fluid extravasation and accumulation in tissues, including the lungs [6]. There is a general consensus that the decline in pulmonary gas exchange is an effect of oxidative stress and tissue injury by metabolites, such as superoxide and peroxynitrite. In concordance, in this study we observed that catalytic degradation of superoxide by R-100 improved pulmonary gas exchange and that there was a tendency, although not significant, toward improvements in tissue injury markers such as lung wet-to-dry weight ratio and histological analyses.

Nitric oxide synthase (NOS) represents a group of enzymes that catalyse NO synthesis. Depending on its location, three main subtypes are defined. Many investigators have described that selective inhibition of NOS isoforms i.e., iNOS or nNOS, shows a beneficial effect on the pathophysiology of ALI [16, 22]. In contrast, another study showed that non-selective inhibition of NOS may be detrimental in severe sepsis [24]. Studies have also reported an improvement in oxygenation in ARDS with NO-inhalation treatment [25]. Excessive NO, especially iNOS-derived NO, has been shown to blunt the pulmonary hypoxic vasoconstriction leading to increased shunt fraction (causing vasodilatation in less ventilated areas) that results in impaired arterial oxygenation. An alternative approach to reduce the shunt fraction is the NO supplementation, assuming that it would increase blood flow in well-ventilated areas, thus limiting the shunt fraction. NO supplementation may also benefit by reducing the pulmonary artery pressure, which is a detrimental complication of ALI leading to increased mortality. In this respect, the exogenous delivery of NO may benefit patients with ALI and sepsis. However, this approach might be detrimental in environments that are associated with severe oxidative stress, including our present model. Therefore, we combined the NO donor with the superoxide degradation catalyst, thus limiting the formation of more toxic product—peroxynitrite. In the present study, R-100 significantly reduced PAP and PCWP as expected although the therapy did not affect increased shunt fraction.

Another important finding in this study was that the fluid balance after 24 h was significantly improved by R-100. Previous studies have shown a massive systemic fluid retention as sepsis progresses [9, 23]. A positive fluid balance is an independent predictor of mortality in ICU patients with pulmonary edema [9, 24]. The improved fluid balance seen in these animals implies less fluid extravasation and accumulation. With respects to pulmonary edema, the histological data from this study also showed that, although not significant, R-100 appeared to decrease tissue injury and fluid accumulation in the alveoli.

The mechanistic aspects behind the beneficial effects of R-100 remain incompletely understood. As mentioned above, the inhibition of excessive NO is beneficial in ALI. However, our present study also suggests that exogenous delivery of NO in combination with a superoxide degradation catalyst may also represent an alternative approach for treatment of ALI. This interesting phenomenon deserves further studies to elucidate the precise mechanism(s) underlying the pleiotropic effects of NO in sepsis. There is also much that is unknown regarding the underlying mechanistic aspects of how R-100 reduces the fluid retention. Future follow-up studies are warranted to elucidate the precise mechanisms. The limitation of the current study is that we were unable to perform a dose-dependent evaluation of R-100. Additionally, we were not able to show data on the oxidative and nitrosative stress. Furthermore our studies were limited to only 24 h. The longer duration may have better illustrated prolonged treatment benefits (i.e., histological analyses and lung wet-to-dry weight ratio).

Nevertheless, treatment with R-100 in sheep subjected to smoke inhalation and *P. aeruginosa* sepsis ameliorates pulmonary hypertension, improves pulmonary gas exchange and reduces the systemic fluid retention.

## Conclusions

R-100 in sheep subjected to smoke inhalation and *P. aeruginosa* sepsis ameliorates pulmonary hypertension, improves pulmonary gas exchange and reduces the systemic fluid retention. Further studies investigating the mechanistical aspects of R-100 are warranted.

## Abbreviations

ALI: acute lung injury
ANOVA: analysis of variance
CFU: colony forming units
CO: cardiac output
COHb: carboxyhemoglobin
CVP: central venous pressure
FiO_2_: fraction inspired oxygen
HR: heart rate
ID: inside diameter
LAP: left atrial pressure
LVSWI: left ventricle stroke work index
MAP: mean arterial pressure
NO: nitric oxide
NOS: nitric oxide synthase
iNOS: inducible nitric oxide synthase
nNOSL: neuronal nitric oxide synthase
ONOO^−^: peroxynitrite
PaCO_2_: arterial partial carbondioxide pressure
PaO_2_: arterial partial oxygen pressure
PvCO_2_: venous partial carbondioxide saturation
PvO_2_: venous partial oxygen saturation
PAP: pulmonary arterial pressure
PCWP: pulmonary capillary wedge pressure
PEEP: positive end expiratory pressure
PMN: polymorphonuclear neutrophil
PVRI: pulmonary vascular resistance index
Qs/Qt: pulmonary shunt fraction
RVSWI: right ventricle stroke work index
SaO_2_: arterial oxygen saturation
SvO_2_: venous oxygen saturation
SCT: single cannula tracheostomy
SEM: standard error of the mean
SOD: superoxide dismutase
SVI: stroke volume index
SVRI: systemic vascular resistance index
